# Recent advances in the treatment of osteoarthritis

**DOI:** 10.12688/f1000research.22115.1

**Published:** 2020-05-04

**Authors:** Susanne Grässel, Dominique Muschter

**Affiliations:** 1 Department of Orthopedic Surgery, Exp. Orthopedics, ZMB/Biopark 1, Am Biopark 9, University of Regensburg, Regensburg, 93053, Germany

**Keywords:** Osteoarthritis, OA phenotype, therapy, inflammation, subchondral bone, cartilage, pain, metabolic syndrome, senescence

## Abstract

Osteoarthritis (OA) is one of the most debilitating diseases and is associated with a high personal and socioeconomic burden. So far, there is no therapy available that effectively arrests structural deterioration of cartilage and bone or is able to successfully reverse any of the existing structural defects. Efforts to identify more tailored treatment options led to the development of strategies that enabled the classification of patient subgroups from the pool of heterogeneous phenotypes that display distinct common characteristics. To this end, the classification differentiates the structural endotypes into cartilage and bone subtypes, which are predominantly driven by structure-related degenerative events. In addition, further classifications have highlighted individuals with an increased inflammatory contribution (inflammatory phenotype) and pain-driven phenotypes as well as senescence and metabolic syndrome phenotypes. Most probably, it will not be possible to classify individuals by a single definite subtype, but it might help to identify groups of patients with a predominant pathology that would more likely benefit from a specific drug or cell-based therapy. Current clinical trials addressed mainly regeneration/repair of cartilage and bone defects or targeted pro-inflammatory mediators by intra-articular injections of drugs and antibodies. Pain was treated mostly by antagonizing nerve growth factor (NGF) activity and its receptor tropomyosin-related kinase A (TrkA). Therapies targeting metabolic disorders such as diabetes mellitus and senescence/aging-related pathologies are not specifically addressing OA. However, none of these therapies has been proven to modify disease progression significantly or successfully prevent final joint replacement in the advanced disease stage. Within this review, we discuss the recent advances in phenotype-specific treatment options and evaluate their applicability for use in personalized OA therapy.

## Introduction

Osteoarthritis (OA) is a multi-factorial, mostly slowly progressing, and primarily non-inflammatory degenerative disorder of the synovial joints that is often age related and/or trauma induced. Degradative processes eventually lead to the irreversible destruction of the articular cartilage and other tissues of the joints. Although OA is the most common musculoskeletal condition worldwide that causes significant health, economic, and social problems, research efforts so far have not been able to define its exact etiology. Age-related wear of articular cartilage and subchondral bone, limb overuse, overloading and mal-alignment, genetic disorders, and metabolic syndromes (obesity, inflammatory responses, and diabetes) are important players in the onset and progression of OA ^1, 2^. For many years, strong and cost-intensive efforts have been undertaken to develop therapies to improve care, quality of life, and pain relief for OA patients. Therapeutic approaches predominantly addressed symptoms and tried to modify/improve structural features of affected joint tissues. Despite this, no therapies have been able to halt or delay OA progression satisfactorily or provided effective and long-lasting symptomatic relief. Currently, joint replacement with an artificial prosthesis is the most effective measure to improve pain sensation and quality of life in patients. The development of novel therapeutic approaches targeting the osteoarthritic degradative and inflammatory processes in cartilage, synovium, or bone requires a deep understanding of the disease status of these joint tissues at the time of the intervention. It is crucial to apply therapies at the early stages of the disease prior to major structural and functional alterations in the osteochondral unit; otherwise, these interventions will not be successful ^3^. Structural and clinical features of OA are characterized by a high interpatient variability. This heterogeneity is considered to be a major factor associated with the complexity of OA and the ongoing difficulties to identify “one size fits all” therapies ^4, 5^.

It is accepted that defining OA subgroups based on risk factors is too simple, so it is of high clinical interest to identify specific OA phenotypes (subgroups of patients with similar clinically observable characteristics, i.e. genetic predispositions combined with environmental factors leading to tibiofemoral OA) and endotypes (disease subtypes defined functionally and pathologically by a molecular mechanism, i.e. different mechanisms leading to the same phenotype as tibiofemoral OA), which are the basis of accurate prognosis and development of personalized therapies ^5^. Several attempts have been made to group and identify OA phenotypes according to pathobiological mechanisms. Felson defines criteria for characterizing OA phenotypes via an epidemiological approach ^4^. He discriminates between generalized arthritis and joint-specific OA, secondary and primary OA, and incident and progressive OA. Another intriguing perspective is presented by Berenbaum *et al*., who provide a novel definition of OA as a “mismatch disease” commonly referred to by evolutionary biologists ^6^. This is defined as a condition that is more common today than in the past because the human body is not well adapted to certain features of modern environments. These features are high levels of physical inactivity, chronic low-grade inflammation, high body mass index, and obesogenic diets (processed foods which are high in sugar and saturated fat and low in fiber content). Other distinctions include the following phenotypes: chronic pain with central sensitization, inflammation, metabolic syndrome, bone and cartilage metabolism, and mechanical and minimal joint disease ^7^. Deveza *et al*. divide according to mechanistic subgroups including senescence, inflammation, pain, and metabolic endotypes ^5^.

However, one thing has to be kept in mind when trying to define subgroups of OA. Although OA can be initiated by multiple factors at multiple sites, mechanical overloading is still the key feature of its pathogenesis. Joint tissues are sensitive to physical stimuli, and OA may result from excessively aberrant or physiologically normal mechanical stresses on initially healthy or pathologically impaired articular cartilage, bone, and ligaments, respectively ^8^. Brandt *et al*. pointed out in a commentary that they feel all OA is secondary due to the accumulation of intra-articular (i.a.) stress and all OA is primarily driven by mechanical stress on the joint ^9^. They postulate a basic mechanical etiopathogenesis for common OA and would rather categorize it on the basis of the underlying mechanical abnormality like post-traumatic, failure to absorb repetitive impulsive loading, and congenital or developmental anatomic incongruities.

This review will select key OA pheno-endotypes according to relevant literature and current clinical trials/therapies that have been the most promising targets for recent clinical or pre-clinical studies.

## Treatments targeting articular cartilage

OA is characterized by the degradation of articular cartilage and bone matrix components. Among the earliest are type II collagen and the proteoglycan aggrecan, leading to the loss of cartilage structure and function ^10, 11^. Cartilage matrix degeneration products are well investigated for drug target discovery. Several key anabolic and catabolic pathway enzymes are dysregulated in OA cartilage, providing the opportunity to identify and validate new drug targets ^12^. To this end, novel combinations addressing existing known targets may be identified. This also includes combinations of therapeutics that are anti-catabolic and those that target anabolic signaling pathways. If investigated, this might lead to identifying novel efficacious add-ons from combining known drugs and targets. For better classification, there should be discrimination between disease-modifying OA drugs (DMOADs), which target pathways of cartilage catabolism and anabolism, and regenerative strategies based on stem cells and their components.

### Anabolic drug: sprifermin

A promising anabolic DMOAD is sprifermin, which is a truncated version of human FGF18 that induces chondrocyte proliferation and cartilage matrix production. The results of a phase Ib study of i.a. injected sprifermin in patients with symptomatic knee OA found a statistically significant dose-dependent reduction in loss of total femorotibial cartilage thickness compared to placebo after 12 months of follow up ^13^. Sprifermin is currently being studied in a phase II multicenter randomized dose-finding clinical study (clinicaltrials.gov: NCT01919164). The i.a. administration of 100 μg of sprifermin to participants with symptomatic radiographic knee OA every 6 or 12 months vs. placebo resulted in an improvement in total femorotibial joint cartilage thickness after a follow up period of 2 years. This improvement was statistically significant, but clinical importance was not clear. Application of a lower dose, 30 μg of sprifermin, every 6 or 12 months vs. placebo did not result in a significant difference and it was uncertain whether the response was long lasting ^14^ ( Table 1A).

**Table 1.** Current OA drug targets addressing several proposed OA phenotypes.

Target	Drug	Trial ID	Affected joint	Results
**A) Treatments targeting cartilage**				
Inhibition of cartilage matrix degradation	MMP-inhibitor PG-116800	NCT00041756	knee OA	Termination due to musculoskeletal toxicity ^12^
Cartilage matrix regeneration	Sprifermin (truncated human FGF18)	NCT01919164	Knee OA	Improvement in total femorotibial joint cartilage thickness ^13, 14^
Cartilage matrix regeneration	BMP-7 or OP-1	NCT01133613, NCT01111045, NCT00456157	Knee OA	Pain improvement in BMP-7 group and placebo group ^15^
**B) Treatments targeting subchondral bone**				
Bisphosphonates/ bone turnover	Zoledronic acid		Knee OA	Reduced BML size and visual analogue scale pain score ^16, 17^
	Risedronate		Knee OA	Reduced pain in patient subgroup with BMLs ^18^
	AXS-02 (disodium zoledronate tetrahydrate)	NCT02746068	Knee OA	Reduced pain in patient subgroup with BMLs ^19^
Inhibition of bone degradation	Cathepsin K inhibitor MIV-711	EudraCT: 2015-003230-26, 2016- 001096-73	Knee OA	Slowdown of bone and cartilage degeneration ^20^
**C) Treatments targeting inflammatory processes**				
IL-1	Anakinra (IL-1 receptor antagonist)	NCT00110916	Knee OA	No improvements of OA symptoms ^21^
	AMG 108 (fully human monoclonal antibody to IL-1R1)	NCT00110942	Knee OA	Minimal clinical benefit ^22^
	Lutikizumab (anti IL-1α/β antibody)	NCT02087904	Knee OA	No improvement in synovitis, minimal effect on WOMAC pain score ^23^
		NCT02384538	Hand OA	No improvement in pain or imaging outcomes ^24^
Tumor necrosis factor-alpha	Adalimumab	ACTRN12612000791831	Erosive hand OA	No effect on pain, synovitis, or BMLs ^25^
			Knee OA	Effective pain reduction, increased physical function ^26^
	Etanercept	NTR1192 (EHOA)	Erosive hand OA	No pain relief, decrease in serum MMP-3 levels ^27, 28^
	Infliximab		Hand OA	In recent-onset RA patients, treatment reduced progression of hand OA ^29^
Toll-like receptor 7/9	Hydroxychloroquine		Hand OA	Failed to show efficacy ^30, 31^
I-kB kinase	SAR113945 (I-kB kinase inhibitor)	NCT01113333, NCT01598415, NCT01511549, NCT01463488	Knee OA	No superior efficacy ^32^
p38 MAP kinase	FX-005	NCT01291914	Knee OA	Pain relief superior to placebo
**D) Treatments targeting pain processes**				
NGF	Tanezumab (anti-NGF antibody)	NCT02697773	Knee and hip OA	Modest improvement in functional and pain scores, safety concerns owing to increased need for joint replacement ^33^
NGF receptor tropomyosin-related kinase A (TrkA)	Pan Trk inhibitor GZ389988	NCT02424942, NCT02845271	Knee OA	Short-term moderate pain reduction compared to control ^34^
Transient receptor potential vanilloid 1 (TRPV1) receptor	Trans-capsaicin (CNTX-4975)	NCT02558439	Knee OA	Intra-articular CNTX-4975 reduced moderate-to-severe pain compared to placebo over 24 weeks ^35^
	Mavatrep (JNJ-39439335)	EudraCT 2009-010961-21	Knee OA	Significant reduction in pain and improvement in function, but dose adjustments required because of altered heat perception and resulting thermal burns ^36, 37^
Kappa-opioid receptor	Selective agonist CR845	NCT02524197, NCT02944448	Knee and hip OA	Dose-dependent pain reduction, more effective in hip OA patients ^38^
Alpha calcitonin gene-related peptide	Galcanezumab (LY2951742)	NCT02192190	Knee OA	Study was terminated owing to inadequate efficacy ^39^
Imidazoline receptor I2	CR4056 (receptor ligand)	EudraCT 2015-001136-37	Knee OA	Successful analgesia, especially in male and overweight patients associated with metabolic syndrome ^40^
**E) Treatments targeting metabolic syndrome**				
Cox-2 and T2DM	Cox-2 inhibitor and metformin	Taiwan National Health Insurance Research Database	Knee OA	Lower rate of receiving joint replacement surgery ^41^
HMG-CoA- Reductase	Statins: simvastatin, atorvastatin, atorvastatin calcium, fluvastatin sodium, lovastatin, nystatin, pravastatin, pravastatin sodium, rosuvastatin, and rosuvastatin calcium	SEKOIA phase III trial	Knee OA	Radiological worsening over 3 years, regardless of other potential confounding factors ^42^
HMG-CoA- Reductase	Statins: atorvastatin, fluvastatin, pravastatin, rosuvastatin, or simvastatin	UK-based Clinical Practice Research Datalink	Hand OA	No protective effect of statins on the risk of developing hand OA ^43^
HMG-CoA- Reductase	Statins: pravastatin, rosuvastatin, simvastatin, fluvastatin, lovastatin, and atorvastatin	Study cohorts: 1. The Malmo Diet and Cancer Study (MDCS) 2. The Malmo Preventive Project (MPP) 3. Swedish Mammography Cohort (SMC) 4. Cohort of Swedish Men (COSM)	Knee or hip OA	Statin use is not associated with reduced risk of consultation or surgery for OA of the hip or knee ^44^

BML, bone marrow lesion; IL, interleukin; IL-1R1, interleukin 1 receptor type 1; MMP, matrix metalloproteinase; NGF, nerve growth factor; OA, osteoarthritis; RA, rheumatoid arthritis; T2DM, type 2 diabetes mellitus; WOMAC, Western Ontario and McMaster Universities Osteoarthritis Index

### Anabolic drug: BMP-7

Another approach was the application of BMP-7, where studies have shown a pro-anabolic effect, making BMP-7 a potential candidate to promote articular cartilage repair. Three clinical trials (two phase I and one phase II) investigated the administration of BMP-7 in patients with knee OA. Two trials were completed without results being posted (NCT01133613 and NCT01111045), and one was completed with results published (NCT00456157) ^15^. There was no dose-limiting toxicity identified in the latter study. Most adverse events were mild or moderate and were similar in placebo and BMP-7 groups. Notably, overall, the outcome of the BMP-7 group was similar to the placebo group, as both groups experienced a 20% improvement in pain ( Table 1A).

### Anti-catabolic drugs: MMP inhibitors

Inhibition of MMPs, i.e. MMP-13 and aggrecanases such as ADAMTS-4 and -5, which are key proteases responsible for cartilage matrix degradation in OA ^45, 46^, might be another way to delay cartilage destruction. A human clinical trial (NCT00041756) involving knee OA patients receiving the MMP inhibitor PG-116800 (PG-530742), which has low affinity for both MMP-1 and MMP-7, was unfortunately terminated because of musculoskeletal toxicity ^12^ ( Table 1A). The most frequent adverse effect was arthralgia (35% of patients), and 13% of patients reported hand adverse events (edema, palmar fibrosis, Dupuytren’s contracture, or persistent tendon thickness or nodules). As a result, MMP inhibitor PG-116800 has not been developed further as a treatment for knee OA ^47^. Other more MMP-13 selective compounds, such as CP-544439, AZD-8955, and WAY-170523, are under investigation, but clinical data have not been released yet ^13^. MMPs and aggrecanases are involved in cartilage matrix degradation, and a balanced activity of these proteases is critical for matrix homeostasis. An unbalanced protease activity favoring rapid cartilage matrix degradation in early OA pathogenesis might classify as an additional OA endotype, which then would qualify as a specific target for drug intervention.

These data demonstrate that compounds such as sprifermin (FGF18) and BMP-7 have promising pro-anabolic effects on cartilage tissue, whereas the inhibition of catabolic factors such as proteases has not shown beneficial effects in cartilage so far owing to adverse effects ( Table 1A).

### Regenerative therapies with stem cells

A total of 144 clinical trials investigating the therapeutic impact of stem cells on OA and on cartilage trauma have been reported to date at www.clinicaltrials.gov, suggesting regenerative medicine may be a promising therapy for future OA management. At first, case reports described the transplantation of mesenchymal stem cells (MSCs) through an invasive approach (surgery). Later, the introduction of autologous MSCs within the joint by i.a. injection, which represents a less-invasive strategy, was reported to be feasible and safe. Indeed, after i.a. injection into SCID mice, MSCs engrafted directly in the injured site, which has been suggested to avoid systemic distribution and toxicity as well as to promote longer survival of the cells ^48^. Currently, mostly autologous MSCs and adipose-derived stem cells (ASCs) are applied for knee OA therapy, but very few approaches are targeting hip OA. Almost all of the trials are phase Ia/b safety and dose-finding studies with a low number of participants. About 45 of the listed studies are completed, but only six of them report results. A total of 55 of the trials that are recruiting or not yet recruiting indicate strongly increasing interest in stem cell therapy not only for traumatic cartilage injury but also for late-stage OA.

### Extracellular vesicles derived from stem cells

Our literature search shows that stem cells, specifically autologous stem cells derived from bone marrow (BMSCs) and adipose tissue (ASCs), are preferred over other cell types for regenerative strategies. However, there is doubt among surgeons and researchers about whether or not stem cells are really the optimal tool for regenerative therapy.

After administration, stem cells tend to disappear quickly from the target tissue; however, their chondroprotective and immunomodulatory effects are long lasting. Presumably, the therapeutic effects are mainly mediated in a paracrine manner, as they appear to be independent of the engrafted cells ^49^. When exposed to inflammatory signals, MSCs develop and show a rich secretory profile. After stimulation of these cells with tumor necrosis factor (TNF)-α, a proteomic approach identified 118 proteins, which were differentially expressed by human ASCs ^50^. However, paracrine effects are not limited to soluble factors, as stem cells and other cell types produce extracellular vesicles (EVs), which are small phospholipid-bilayer-enclosed particles carrying many cytoplasmic components ^51, 52^. EVs play a role in a number of different cellular activities, such as communication between cells via horizontal transfer of mRNA and proteins, and they are distinguished according to size. EVs are attractive options for therapeutic use because of their unique physical and biological characteristics, which include high biocompatibility and intrinsic targeting activity ^53^. Exosomes, the most-studied group of EV, can be as small as 30–150 nm in diameter, so it may be possible for them to passively diffuse through tissues ^54^. Overall, the consensus is that stem cell secretomes and EVs applied for the treatment of cartilage pathology and knee OA had pleiotropic and overall positive effects ^55^. *In vitro*, anti-catabolic, immunomodulatory, and regenerative properties were assigned to the secretome and EVs. Pre-clinical *in vivo* studies resulted in positive effects on the joint and confirmed the effectiveness of EV injections as a minimally invasive therapy ^56^. Exosome injections partially improved the gait abnormality patterns in an OA mouse model ^57^, and MSC secretome injections provided early (day seven) pain reduction in treated mice ^58^. All together, these data support the translational potential of this regenerative approach. The promising *in vitro* and *in vivo* results support the potential of this new treatment strategy, opening up new perspectives for cell component-based therapies. EVs are proposed as next-generation biomarkers to predict the pathophysiological state of the joint ^55^, assigning an important role for EVs in future therapies for the treatment of joint disorders. Remarkably, they constitute a simpler, and most of all safer, alternative to actual cell-based therapeutic strategies, as they are cell derived but not living cells and thus cannot proliferate or form tumors. As known for cells, EVs can also be combined with scaffolds, either bound on their surface or embedded within the biomaterial matrices. Specific activation signals such as ultrasound may enable the controlled release of specific subpopulations of EVs, i.e. exosomes.

## Therapies addressing subchondral bone

Besides nutrient supply and metabolism, physiological and non-physiological shock absorption and support of overlying cartilage are the main functions of subchondral bone ^59, 60^. Therefore, any changes affecting bone cell metabolism, structural integrity, and architecture might render the bone more susceptible to aberrant loading or even induce abnormal reactions to normal physiological load. OA-related changes in subchondral bone structure were long regarded as an adaptation of bone to the biomechanical changes observed in articular cartilage. Recently, several pre-clinical and clinical studies demonstrated that alterations in bone structure might even precede and instead mediate cartilage pathology ^61, 62^ and that OA progression is associated with temporal changes in bone structure ^60^. In early OA, accelerated bone turnover leads to bone plate thinning and increased porosity, whereas the trabecular compartment shows increased trabecular spacing and decreased bone volume fraction. Progression of OA is accompanied by subchondral bone plate thickening, increased trabecular thickness, and increased bone volume fraction ^60^. Bone marrow lesions (BMLs), a hallmark of OA, appear early on MRI and are associated with increased pain and cartilage degeneration ^63^.

### Therapies with bisphosphonates

Bisphosphonates (BPs) effectively slow down bone turnover by inhibiting osteoclast activity in osteoporosis, but their usability in OA remains uncertain ^18^. There are indications that a specific patient subgroup might respond to BP use: intravenous zoledronic acid successfully reduced BML size and visual analogue scale (VAS) pain score after 6 months in a randomized controlled trial, though a second multicenter trial could not confirm the results ^16, 17^. Furthermore, a meta-analysis by Vaysbrot *et al*. identified similar effects in a trial using oral risedronate treatment in a patient subgroup with BMLs ^18^. An active phase III trial (NCT02746068) using AXS-02 (disodium zoledronate tetrahydrate) in knee OA with associated BMLs provided promising results in the reduction of pain supposedly by suppression of local acid and pro-inflammatory cytokine production ^64^. The ongoing phenotype debate in OA raised the question of whether the effectiveness of BPs has been confounded owing to the heterogeneity of the patient group enrolled in clinical trials so far ^64^. Indeed, BPs might be especially beneficial in patients with BML or high bone turnover in an early state of OA. Interestingly, pharmacologic agents like BPs that directly affect osteoclast activity effectively reduced pain. Recent research identified osteoclasts as the inducers of OA bone pain by induction of sensory innervation in a murine OA model ^65^. Determination of more of these connections and more precise categorization of patient subgroups (like bone pain due to BML development or increased osteoclast activity/bone turnover) might lead to the repurposing of already existing drugs for new targets.

### Drugs targeting bone cells

New therapeutic approaches include neutralization of cathepsin K, the major osteolytic protease released by osteoclasts. The “small molecule” cathepsin K inhibitor MIV-711 effectively attenuated joint pathology in a rabbit OA model ^66^ and slowed bone and cartilage degeneration in a phase IIa multicenter trial of primary knee OA ^20^. With 26 weeks’ duration, the study was relatively short, and MIV-711 did not reduce pain during this time ( Table 1B). Denosumab, a monoclonal antibody directed against RANKL and thereby inhibiting osteoclastogenesis, is currently tested in erosive OA of interphalangeal finger joints (NCT02771860) and in knee OA (DISKO, ISRCTN96920058), but results have not been published to date. Potential new targets to address subchondral bone include TGFβ, which is elevated in OA synovial fluid ^67^. Systemic blocking of TGF prevented bone and cartilage degeneration in a rodent OA model ^68^, but targeting this specific molecule needs to take into consideration the physiological role of TGFβ as a differentiation stimulus for chondrocyte precursor cells ^67^. Furthermore, OA bone is targeted by anabolic therapies. Teriparatide, a synthetic parathyroid hormone, effectively reduced chondrocyte apoptosis and attenuated OA progression after i.a. application in a surgical rat OA model ^69^. Its effectivity is currently being evaluated in a phase II trial of knee OA (NCT03072147).

### Dietary supplementation of vitamin D _3_

Additional dietary supplementation of vitamin D _3_ (cholecalciferol) might be an option to target and strengthen bone in OA owing to its ability to increase calcium and phosphate uptake from the gut and its direct effect on bone cell metabolism ^70^. Numerous trials for vitamin D _3_ supplementation in OA patients can be found at clinicaltrials.gov, but there are contradictory reports about a relationship between vitamin D levels and a higher risk for OA incidence and progression ^71^. A meta-analysis of randomized controlled trials revealed a reduction in WOMAC pain and improved joint function in OA patients after vitamin D _3_ intake, but only at a concentration of 2,000 IU ^72^. Cartilage degradation was not affected. Generally, vitamin D _3_ intake might be beneficial for a large proportion of the population, as its deficiency is a worldwide problem and elderly people, who are also at an increased risk of OA, are often affected ^73^.

Restoration of bone metabolism and structure might be a worthwhile goal because of the huge importance of this structure as a mechanic buffer for proper load perception and distribution. A detailed knowledge of timely changes in OA-related bone metabolism might enable a more precise use of bone anabolic and anti-catabolic therapies to restore or prevent bone degradation. Maintenance of bone structure and shock-absorbing abilities might prevent cartilage alterations and therefore put a hold on subsequent degradative events culminating in joint failure.

## Treatments targeting inflammatory mediators and pathways

It is now commonly accepted that OA has an inflammatory component that might be more dominant in specific patient subgroups and joint tissues. The release of various pro-inflammatory mediators like prostaglandins, cytokines, and chemokines has been demonstrated in numerous pre-clinical OA animal models and in patients ^74^. Synovitis is a common feature of inflammatory OA, and technical progress in imaging technologies like ultrasound and MRI revealed synovitis in a large number of patients at different disease stages ^75, 76^. A plethora of triggers including aberrant mechanical forces, metabolic syndrome, increased age, and fragments of cartilage extracellular matrix (ECM) or crystals might induce the release of these mediators from various responsive joint tissue cell types. A large number of recent review articles address cells and components of the innate immune system as the main drivers of OA inflammatory processes ^77, 78^. Non-steroidal anti-inflammatory drugs and glucocorticoids are commonly used to treat OA but are not optimal owing to moderate effectiveness and serious side-effects in long-term use ^79, 80^.

### Anti-cytokine therapy

Most biologics used to treat OA-related inflammation were developed for rheumatoid arthritis (RA), a disease associated with more pronounced inflammation. So far, biologics targeting the inflammatory cytokines interleukin (IL)-1 and -6 as well as TNF-α have not been useful in the prevention of pain or structural progression of OA ( Table 1C). Granulocyte/macrophage colony-stimulating factor (GM-CSF) was associated with inflammatory pain in a mouse OA model ^81^ and was associated with hip OA over knee OA in a study analyzing mononuclear cell contribution to synovial inflammation ^82^. The anti-GM-CSF antibody otilimab (GSK3196165) was enrolled in a study for erosive hand OA and yielded promising results (NCT02683785) ^83^. Mavrilimumab, a GM-CSF receptor inhibitory antibody effective in RA ^84^, might therefore also have potential as an OA drug. The predominantly low-grade and non-systemic inflammation observed in OA might explain the limited success of single cytokine blockade. Concentration on specific OA subsets like erosive hand OA associated with more pronounced inflammation potentially presents a responsive patient group for anti-cytokine biologics. Identification of a single potent driver of OA inflammation seems to be difficult, and broader approaches targeting general pro-inflammatory signaling pathway components might be more favorable ( Table 1C).

### Interference with pro-inflammatory signaling pathways

Targeting single cytokines has so far had little effect. Recent strategies aim to interfere with further upstream initiators of the pro-inflammatory signaling cascade. Hydroxychloroquine (HC), a chloroquine derivative used to treat malaria and inflammatory autoimmune disorders like RA, supposedly exerts its effects via Toll-like receptor (TLR) 7/9 ^85^. Several studies using HC in hand OA have failed to show effects so far ^30, 31^, and results from a study in knee OA are not obtainable yet (NCT01645176). TLR downstream targets include molecules like MyD88, TRAF3/6, p38 MAPK, Janus kinases, and transcription factors like NF-κB ^86^. Several attempts have been made to interfere with signaling molecules to inhibit inflammation in OA. The I-κB kinase inhibitor SAR113945 was tested in four trials of knee OA (NCT01113333, NCT01598415, NCT01511549, and NCT01463488), but, though the compound showed a good safety and tolerability profile, a larger proof-of-concept study failed to show superior efficacy ^32^. A small subgroup of patients with effusion at baseline elicited a reduced WOMAC pain score after 56 days. Local delivery of a potent p38 MAPK inhibitor (PH-797804) reduced joint destruction and inflammation in a murine destabilization OA model ^87^. The efficacy of PH-797804 compared to naproxen was evaluated in a clinical trial involving knee OA patients, but the results have not yet been disclosed (NCT01102660). FX-005, another therapeutic p38 MAPK inhibitor with sustained-release kinetics, was evaluated in a phase I/II knee OA trial where it promoted pain relief superior to placebo after 4 weeks (NCT01291914) ( Table 1C). Direct targeting of the TLR would provide even higher upstream interference with OA immune activation, e.g. the application of a miR-21 inhibitor targeting TLR7 was able to induce long-lasting analgesia in an OA rat model ^88^. As for anti-cytokine therapy, careful evaluation of individual patient inflammatory status will probably help to identify more responsive patient subgroups or joints.

### Other immune system targets

The complement system might also be a potential source of therapeutic targets for OA therapy ^89, 90^. An increasing number of drugs targeting different factors of the complement cascade are available and were tested in the clinic for various diseases ^91^, but, to the best of our knowledge, none of them has been tested in OA patients so far. Similarly, there is increasing awareness that adaptive immune mechanisms might be involved in OA pathophysiology ^92, 93^, but these insights have not been translated into therapeutic approaches so far. Identification of more immune system entities contributing to OA development and progression will provide even greater numbers of targets for potential OA therapeutics but requires meticulous analysis of timely and spatial involvement in disease-related processes.

### Gene therapies

Novel genetic approaches are currently under evaluation for safety in clinical phase I studies. They include i.a. injection of recombinant adeno-associated virus type 2/5 (rAAV2.5) vector encoding IL-1 receptor antagonist (IL-1Ra) into one knee joint of patients with moderate OA of the knee (NCT02790723) and FX201, a helper-dependent non-integrating adenovirus, containing the human IL-1Ra gene under the control of an inflammation-sensitive promoter (NCT04119687). Moreover, new gene therapeutic targets include interferon (INF)-β (rAAV2.5 vector encoding human IFN-β under control of a NF-κB promoter, ART-I02) in subjects with RA or OA and active arthritis of the hand (NCT02727764) as well as XT-150, a plasmid DNA carrying a variant of the human IL-10 transgene (NCT03477487). Although results of these studies are not available yet, gene therapy offers great therapeutic potential as it is aimed at prolonged, site-specific, and controlled release of treatments in the target joints. Confining anti-cytokine or any anti-inflammatory treatment to specific joints could potentially prevent the side-effects observed with a more classic biologic treatment plan ^94^. Virus-related delivery systems might provide superior performance over classical delivery systems but, clearly, safety aspects (e.g. immunogenicity of the vector, off-target and long-term effects) have to be taken seriously and analyzed carefully in relation to effectiveness before considering any kind of genetic therapy ^95^. In general, data from an equine study using AAV2.5-delivered IL-1Ra showed promising results regarding pharmacodynamics and safety profile ^96^. As mentioned above, first-in-human clinical trials will evaluate the safety profile of gene-related therapies and will give a general hint regarding the applicability of gene therapies for OA.

## Treatments addressing pain

Apart from structural deterioration in OA joints, pain is a dominant and probably the most debilitating hallmark of OA pathology and the *a priori* reason why patients see a physician. Huge effort has been put into OA-related pain research to identify underlying mechanisms, but, because of its complexity, no general guidelines could be identified for its effective treatment ^97, 98^. The sensation of pain in OA does not show uniform appearance among patients and during progression. The source of OA pain includes nociceptive pain komma inflammatory pain and neuropathic pain as well as processes of peripheral and central sensitization. Structural features like BMLs, synovitis, and joint effusion show a strong association with pain intensity ^63, 99, 100^.

Classical treatments include the use of acetaminophen (paracetamol), NSAIDs, and opioids, which induce a plethora of unwanted side effects. Here komma we will discuss the latest developments in therapies directly targeting neuronal structures to alleviate OA pain.

### Anti-nerve growth factor antibody treatment

A huge effort has been made in the development of therapeutics targeting nerve growth factor (NGF). NGF might be released upon mechanical stimulation or inflammatory mediators from different cell types including osteoclasts, osteocytes, chondrocytes, synovial fibroblasts, and macrophages in pre-clinical OA studies and human OA ^100– 105^. After the first promising clinical tests using anti-NGF antibodies, the FDA stopped ongoing trials owing to reports of serious adverse side effects with fast progression of OA and increased demand for knee replacement surgery. After the identification of risk factors (NSAID use) and dose modifications, the hold was lifted in 2015 and clinical trials continued (extensively reviewed in 106, 107). A new phase III trial of knee and hip OA using subcutaneous injections of tanezumab (monoclonal anti-NGF antibody) displayed modest improvements in pain and functional scores compared to control but again raised safety concerns after increased need for total joint replacement in the tanezumab group ^33^. Active immunization against NGF might provide a new alternative to target chronic pain, as demonstrated in murine OA ^108^. Although initial trials using anti-NGF antibodies looked promising, further studies are needed to warrant treatment safety.

### Anti-nerve growth factor receptor strategy

New strategies to inhibit NGF-induced pain concentrate on the antagonization of its receptors tropomyosin-related kinase A (TrkA) and p75 ^NTR^. TrkA inhibition effectively reduced pain behavior in several pre-clinical OA models ^109, 110^. Various molecules have been developed to target the TrkA receptor: pan Trk inhibitor GZ389988 (NCT02424942 and NCT02845271), AR786 (allosteric selective TrkA inhibitor), ASP7962 (TrkA receptor antagonist, NCT02611466 ^111^), ONO-4474 (pan Trk inhibitor), and VM902A (allosteric TrkA selective inhibitor). Only GZ389988 exhibited modest pain reduction compared to control after 4 weeks but no prolonged efficacy after 12 weeks ^34^ ( Table 1D). NGF receptor p75 ^NTR^ concentration is increased in the blood, synovial fluid, and tissue macrophages of OA patients ^112^ and has been related to inflammatory pain in a rat model ^113^. LEVI-04, a p75 neurotrophin receptor fusion protein (p75NTR-Fc), is currently being investigated in a phase I clinical trial (NCT03227796), but results are not available yet.

### Ion channels

Furthermore, ion channels like transient receptor potential (Trp) and Nav1.7/Nav1.8 voltage-gated sodium channels gained attention as potential drug targets ^114, 115^. TRP vanilloid 1 (TRPV1) channels expressed by sensory neurons might be targeted using receptor agonists like capsaicin or resiniferatoxin. In various animal models, these molecules resulted in long-term desensitization or nerve degradation, thus reducing pain and neuropeptide release, but conflicting observations have been reported ^114^. Currently, several active clinical trials are analyzing the use of topical and i.a. trans-capsaicin (CNTX-4975) in knee OA. Stevens *et al*. described a dose-dependent effect of i.a. CNTX-4975 that reduced pain compared to placebo over 24 weeks in patients with moderate-to-severe knee OA ( Table 1D) ^35^. The FDA assured Fast Track designation to CNTX-4975 for the treatment of OA knee pain in 2018. Several “small molecule” TRPV1 antagonists have been tested so far in healthy subjects but not in OA pain ^116, 117^, and some had to be stopped because of inefficiency or adverse effects, including NEO6860, which caused headache, nausea, fatigue, and increased blood pressure among other adverse effects ^118, 119^. A single dose of JNJ-39439335 (Mavatrep), a selective competitive TRPV1 receptor antagonist, was evaluated in phase I studies and successfully reduced pain and improved functional score in knee OA patients after 7 days, though future studies require dose adjustment owing to adverse events involving thermal perception ^36, 37^ ( Table 1D). Animal models indicate involvement of voltage-gated sodium channels Nav1.7 and Nav1.8 in pathological pain states ^120^, and A-803467, a selective Nav1.8 channel-blocking agent, disrupted nociceptive signal transmission in monoiodoacetate (MIA)-induced OA ^121^. Furthermore, Vertex’s sodium-channel blocker VX-150 showed efficacy in acute pain reduction and was granted a breakthrough therapeutic potential by the FDA, though results of a completed phase IIa clinical study have not been published yet (NCT02660424) ^122^.

### Targeting peripheral opioid receptors

Opioids are effective analgesics but their use is limited due to serious adverse side effects like constipation, respiratory depression, tolerance, and dependence. Currently, new opioid receptor (OR) agonists with an improved safety profile targeting the µ, δ, and κ subtypes are in development. Selectively targeting the peripheral κ-OR might avoid side effects observed when drugs target the µ-OR. Cara Therapeutics developed the selective κ-OR agonist CR845 and evaluated its efficacy in knee and hip OA (NCT02524197 and NCT02944448), reporting dose-dependent efficacy in the reduction of pain in hip OA over knee OA ^38^ ( Table 1D).

### Evolving new targets

Pain relief might also be achieved by targeting the nociceptin/orphanin FQ peptide receptor (NOP) ^123^. Cebranopadol (GRT6005), a dual agonist for the NOP/µ-OR, proved to be safe and efficient in chronic back pain ^124^ and was recently tested in patients with painful knee OA (NCT01357837 and NCT01709214). Other nerve-associated targets include the bradykinin B2 receptor (Fasitibant, NCT02205814 and NCT01091116) and the CB2 cannabinoid receptor (GW842166 [NCT00479427 and NCT00447486], LY2828360 [NCT01319929]). Direct blockade of the sensory neuropeptide α-calcitonin gene-related peptide using LY2951742 (galcanezumab) failed to reduce pain in patients with mild-to-moderate OA ^39^ ( Table 1D). CR4056, an imidazoline-2 ligand with powerful analgesic properties, inhibited inflammation-induced PKCε phosphorylation and membrane translocation in sensory neurons ^125^ and effectively reduced allodynia and hyperalgesia in two rat OA models ^126^. A recent phase II trial in knee OA patients reported successful analgesia, especially in male patients and in overweight patients associated with the metabolic phenotype ^40^ ( Table 1D).

Despite huge efforts invested in the development of new OA analgesics and although several candidates look promising and more and more potential drug targets are identified, pain reduction in OA is still relatively unsuccessful. The complex and diverse underlying mechanisms of OA pain, the timely and spatial alterations of pain types and sensitization, and the interaction of nerves and OA-related structural changes, immune reactions, and altered metabolic conditions still require more intense interdisciplinary research to achieve effective pain management.

## Metabolic syndrome therapies related to OA

Metabolism can be altered in OA, and there are multiple metabolic components underlying metabolic dysregulation. The metabolic syndrome, characterized by excessive and long-term positive energy balance, is defined by several cardio-metabolic factors that commonly are found together with obesity, which are central adiposity, dyslipidemia, impaired fasting glucose levels, and hypertension. People with metabolic syndrome are prone to developing a variety of disorders, especially cardiovascular diseases, type 2 diabetes mellitus (T2DM), and some forms of tumor. The increase in prevalence of metabolic syndrome in industrialized nations, and an association with obesity, together with the fact that it was a rare disease in pre-industrial populations, leads to the hypothesis that the metabolic syndrome might be a major risk factor for OA nowadays ^6, 127, 128^.

Besides hypertension, which seems to provide an elevated risk of knee OA ^129^, T2DM and knee OA often coexist and are known for common risk factors such as obesity and aging. The mechanical impact of excess body weight on joints may explain lower limb OA. However, it is unclear whether T2DM is linked to OA independently of excess weight and whether T2DM is involved in OA pathology. A coexistence between the occurrence of T2DM and OA was shown, but a causal link is not yet established ^130^. T2DM clearly has an unhealthy effect on OA via two pathways: (1) chronic hyperglycemia, which is connected to oxidative stress, abnormal production of pro-inflammatory cytokines and advanced glycation end products (AGEs) in joint tissues, and (2) insulin resistance, which may have local effects but may also maintain a systemic low-grade inflammatory state ^131^.

### Metabolic targets for osteoarthritis therapy

So, are there metabolic targets known that are suitable for OA therapy? Some experimental studies show that mTOR signaling pathways can activate autophagy, which might be an effective approach for treating OA ^119^. OA chondrocytes where AMP kinase (AMPK) has been removed exhibit increased catabolic responses to pro-inflammatory cytokines and biochemical injury. These effects are attenuated by molecules that activate AMPK, indicating that decreased AMPK activity is associated with cartilage degradation ^120, 121^. Possibly, AMPK-activating drugs such as methotrexate, metformin, and sodium salicylate might be good candidates to combat OA progression. A Taiwanese study examined whether or not the use of a COX-2 inhibitor with metformin in OA patients with T2DM was related to fewer joint replacement surgeries than the use of a COX-2 inhibitor only ^122^. At the end of a 10-year follow up period, fewer joint replacement surgeries seemed to be needed in the case group compared to the control group. This outcome may be attributed to the fact that a combination therapy decreases pro-inflammatory factors associated with OA progression much more than one without metformin therapy ( Table 1E).

### Statin usage and osteoarthritis

Some pre-clinical and clinical data are available regarding the effects of statin usage on OA progression. Farnahgi *et al*. aimed to define the effects of hypercholesterolemia on the progression of OA in a murine OA model ^132^. Surgical destabilization of the medial meniscus in knees from mice which were fed a high-cholesterol diet compared to controls led to a severe increase in OA symptoms. Doses of free cholesterol as recommended clinically resulted in overproduction of reactive oxygen species (ROS) and mitochondrial dysfunction. Hypertrophic and degradative markers were upregulated in chondrocytes, resulting in increased breakdown of the cartilage matrix. The authors reported that the severity of these diet-induced OA symptoms was reduced by the application of atorvastatin and a mitochondrial-targeting antioxidant, thus implicating that hypercholesterolemia promotes OA progression by mitochondrial dysfunction in chondrocytes, which was in part a result of increasing ROS production and apoptosis.

In a post-hoc analysis of the SEKOIA trial, the impact of statin use on radiological progression in patients with radiological and symptomatic knee OA was investigated. Results demonstrated that the use of statins was related to radiological deterioration over the course of 3 years irrespective of other potentially related factors, such as obesity or T2DM hypertension, disease duration, symptom intensity and radiological severity ^42^. Another trial investigated the association between statin therapy initiation and incidence of hand OA, but no association was observed in this study ^43^. A pooled analysis based on time-to-event analysis of four population-based large cohorts demonstrated that statin use is not associated with reduced risk of consultation or surgery for OA of the hip or knee ^44^ ( Table 1E).

It appears that repurposing some drugs such as metformin might identify valuable candidates for the treatment of OA in the context of metabolic syndrome. However, clinical studies assessing the effect of other compounds, such as statins, on knee OA progression have shown conflicting results. In line with this, more data and a priori clinical studies are necessary to correlate unambiguously the increase of metabolic syndrome in modern times with OA.

## Therapies targeting senescence and aging

Age is a key risk factor for the development of OA, and age-related changes within the joint might represent targets for therapy. Aging and OA are closely related but still occur independently of one another. Some hallmarks of aging can influence the development of OA, such as genomic instability, telomere attrition, epigenetic alterations, loss of proteolytic homeostasis, dysregulated nutrient sensing, mitochondrial dysfunction, cellular senescence, stem cell exhaustion and altered intercellular communication ^137, 138^. So far, no human clinical trials have been designed to specifically target aging-related processes, but pre-clinical studies targeting some of the age-related factors generated promising data which may lead to novel therapeutic strategies. In the meantime, there are several “senolytics” known, which have the potential to qualify as therapeutic agents to treat OA.

### Cell cycle inhibitors

Cellular senescence leads to the senescence-associated secretory phenotype (SASP), which is characterized by cell-cycle arrest, enhanced production of pro-inflammatory cytokines and other factors ^138^, and increased expression of the cell cycle inhibitor p16 ^Ink4a^ ^139^. Baker *et al*. addressed the physiological relevance and effects of naturally occurring senescent cells. The authors injected a transgene, INK-ATTAC, which was previously established in their lab, into p16 ^Ink4a^-expressing cells of wild-type mice to induce apoptosis ^140^. Their study did not address OA specifically, but they convincingly demonstrated that p16 ^Ink4a^-positive cells accumulate during adulthood and have a detrimental effect on lifespan and encourage age-dependent alterations in various organs. Removing these cells could offer an attractive approach to healthy lifespan extension in joint tissues, which overexpress this cell cycle inhibitor. A direct connection to OA was tested by using the p16-3MR transgenic mouse, which harbors a p16 ^INK4a^ (Cdkn2a) promoter ^141^. Introducing OA in this mouse strain after anterior cruciate ligament transection (ACLT) allowed the selective following and removal of senescent cells. I.a. injection of a senolytic molecule, UBX0101, which selectively kills senescent cells, attenuated the development of post-traumatic OA, reduced pain and the production of SASP factors, and improved the development, phenotype, and function of human OA chondrocytes in 3D pellet culture. The half-life of UBX0101 was low, preventing systemic exposure, but was still effective in blocking cartilage degradation by eliminating senescent cells rather than blocking their SASP secretion.

Targeting cell cycle inhibitors appears to be an intriguing new strategy to halt OA progression by addressing a risk factor, aging, that is closely associated to OA.

### Redox signal pathways and osteoarthritis

An important therapeutic target might be redox-signaling pathways and associated mitochondrial dysfunction in OA. It is accepted that increasing levels of ROS contribute to age-related diseases by promoting cellular dysfunction and abolishing physiological cell signaling pathways ^142^. The prevention of mitochondrial peroxiredoxin (PRX) 3 hyperoxidation-induced expression of mitochondrial catalase abrogated p38-mediated cell death and restored homeostatic signaling to maintain the viability of aging chondrocytes ^143^. Another promising target could be superoxide dismutase 2 (Sod2), as deletion of Sod2 enhanced the severity of OA in mice ^144^. Another interesting target for counteracting oxidative stress-induced tissue damage might be nuclear receptor erythroid 2 related factor (Nrf2). Nrf2 is a key transcription factor that regulates the expression of phase II antioxidant enzymes. These enzymes protect against oxidative stress and tissue damage. Cai and colleagues explored the role of Nrf2 in OA pathogenesis and the effects of Nrf2 acetylation for histone deacetylase inhibitor (HDACi) protection. For this, they took advantage of two murine OA models: i.a. injection of MIA and destabilization of the medial meniscus (DMM) ^145^. In order to analyze the efficacy of HDACi on protection from cartilage damage, a pan-HDACi, trichostatin A (TSA), was applied. TSA promoted the induction of Nrf2 downstream proteins in mouse joint tissues and reduced the expression of OA-associated proteins like several MMPs and pro-inflammatory cytokines. TSA markedly ameliorated the cartilage damage in both OA models but offered no significant protection in Nrf2-knockout mice, suggesting that the protective effect of HDACi on OA progression was Nrf2 dependent.

Addressing redox-signaling pathways and mitochondrial dysfunction will enable exciting novel strategies to combat cellular senescence in general and thereby eliminate a major risk factor for OA: age.

Antioxidants may also have bone-protective effects in OA pathology. Using a spontaneous OA model, the STR/Ort mice, Javaheri *et al*. treated these mice for 3 months with SFX-01®, a synthetic stable variant of sulforaphane, a naturally occurring antioxidant ^146^. SFX-01® treatment both modifies bone architecture in the STR/Ort mice and likely reduces OA pain and improves gait without improving articular cartilage lesion severity and occurrence of osteophytes in the joints of these mice. These findings strengthen the possibility that bone-targeting therapies with antioxidants may have some merit and exert osteotrophic effects possibly not only in OA.

## Conclusions and open questions

For a few years, a novel concept considered OA as a multi-faceted disease involving the whole joint and not only cartilage or synovium. This offers new options to identify and develop novel therapeutics and to re-profile candidate drugs. Recent advances in OA pathology have enlightened key roles of several new pathways, which can be targeted. However, as OA is a highly heterogeneous disease, a single therapeutic targeting a single joint tissue may not be effective and no “one size fits all” drug/therapy will ever be developed. Improved patient stratification in combination with advanced DMOADs and cell-based therapies might lead to the development of personalized OA therapeutics.

Attention to temporal changes in disease progression like the transition from high bone turnover in early OA to decreased bone turnover in the late stages or timely changes in the pain type requires precise knowledge of the underlying mechanistic alterations. Choosing appropriate medication for selective disease time-points might help tailor individual treatment regimens for each patient in the future. Additionally, OA might present itself with overlapping endotypes like, for instance, an inflammatory pain endotype that could benefit from a combination of pharmaceuticals addressing both pain and inflammation.

Many clinical studies have been conducted in OA that address mainly structural targets like cartilage and bone in combination with reduction of inflammation and pain. In general, success was marginal, and only very few drugs, i.e. sprifermin or some BPs, resulted in improvement of joint structure and function. In addition, targeting TrkA or TRPV1 led to pain relief; however, no pharmacological treatment was able to halt or reverse OA progression long term.

Data from the application of MSCs/ASCs generated some guarded optimism; however, so far, only cartilage lesions were addressed with cell-based therapies, whereas subchondral bone, tendons, and other joint tissues were not included. This is a crucial shortcoming, as OA is recognized as a whole-joint disease.

**Figure 1.**
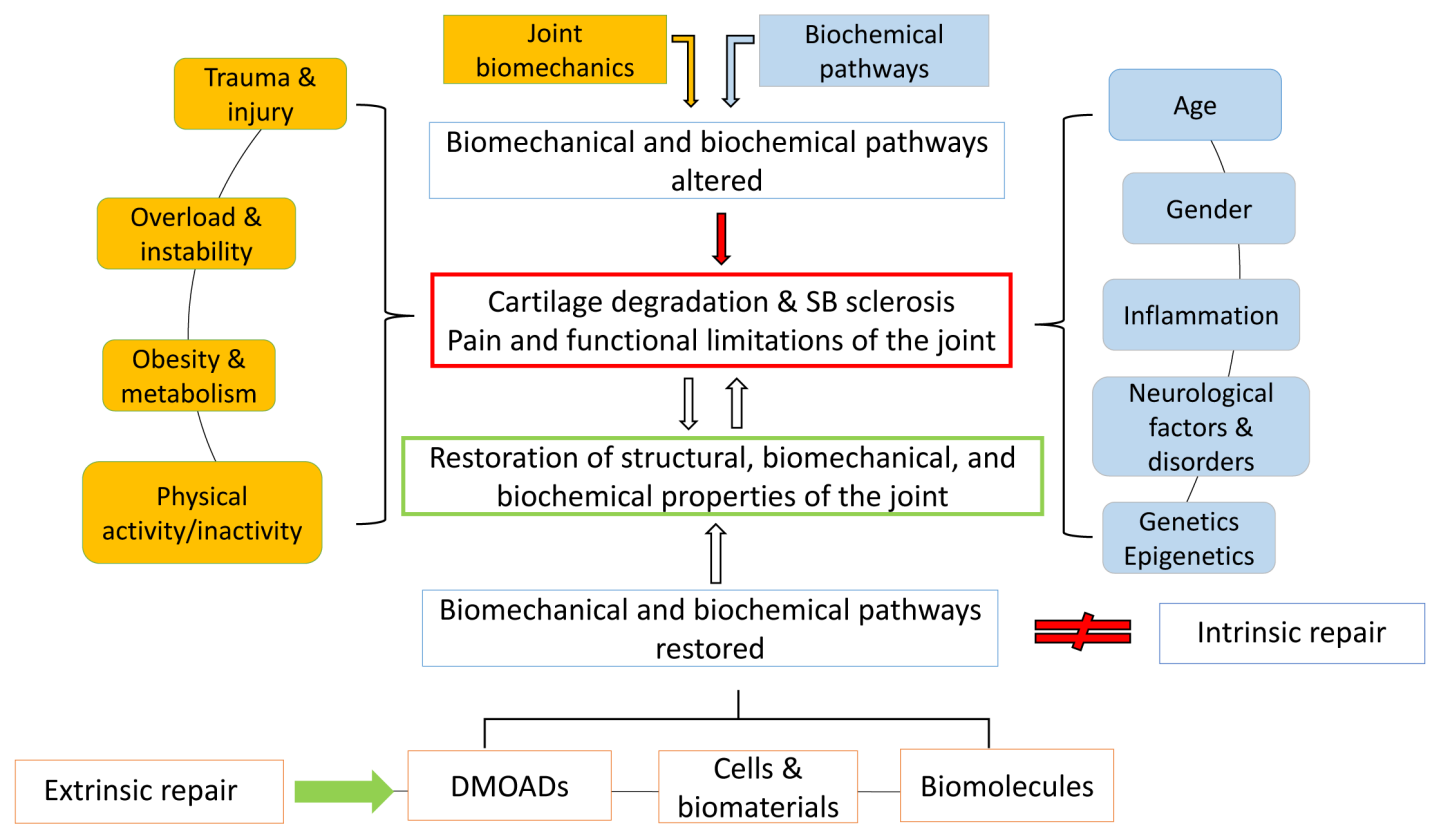
Critical factors for the pathogenesis of OA. Intrinsic repair mechanisms are limited and therefore extrinsic repair support is required to restore or ameliorate joint function. DMOAD, disease-modifying osteoarthritis drug; SB, subchondral bone.

One central point to be considered for all future therapeutic approaches, either regenerative or pharmacological, is the mechanical status of the joint. With this in mind, it is strongly suggested that the altered joint mechanics that cause OA are addressed in a first-line therapy. If altered OA joint mechanics are not normalized and original biomechanical pathways are not restored, it will be most likely that pharmacological or biological treatments of articular cartilage or inflammatory processes will not be efficacious. Furthermore, modulation of derailed cellular mechanoreceptive pathways might provide new opportunities to halt structural tissue deterioration. OA is not a single disease with a common pathophysiological pathway, as many pathways and risk factors lead to mechanical failure of the joint ( Figure 1), so identifying early OA stages would certainly be advantageous for the development of more efficient, targeted therapies. Therefore, the identification of reliable biomarkers and even more advanced imaging methods as well as stronger inter-disciplinary treatment regimens is indispensable.

Considering these central points, personalized OA therapy is the ultimate goal, and recent advances in phenotype classification and targeted drug development might provide a pool of suitable therapeutic options in the future.
